# Phase I study of NT-I7, a long-acting interleukin-7, in severe treatment-related lymphopenia following standard radiation and temozolomide for high-grade glioma

**DOI:** 10.1093/noajnl/vdaf117

**Published:** 2025-06-07

**Authors:** Jian L Campian, Stuart A Grossman, Angela Shaulov Kask, Samuel Kosydar, Roy Strowd, Anna Piotrowski, Justin Tang, Milan G Chheda, John F DiPersio, Dan Schullery, Leonard D’Amico, Serena Desideri, Neeraja Danda, Sara Ferrando-Martinez, Byung Ha Lee, Steven P Fling, Xiaobu Ye

**Affiliations:** Department of Neurology, Mayo Clinic, Rochester, Minnesota, USA; Sidney Kimmel Comprehensive Cancer Center at Johns Hopkins, Baltimore, Maryland, US; Fred Hutchinson Cancer Center, Seattle, Washington, USA; Department of Neurology, Mayo Clinic, Rochester, Minnesota, USA; Wake Forest University School of Medicine, Winston-Salem, North Carolina, USA; Memorial Sloan Kettering Cancer Center, New York, New York, USA; Department of Neurology, Mayo Clinic, Rochester, Minnesota, USA; Department of Medicine, Division of Oncology, Washington University, St. Louis, Missouri, USA; Department of Medicine, Division of Oncology, Washington University, St. Louis, Missouri, USA; Fred Hutchinson Cancer Center, Seattle, Washington, USA; Fred Hutchinson Cancer Center, Seattle, Washington, USA; Sidney Kimmel Comprehensive Cancer Center at Johns Hopkins, Baltimore, Maryland, US; Sidney Kimmel Comprehensive Cancer Center at Johns Hopkins, Baltimore, Maryland, US; NeoImmuneTech, Inc., Rockville, Maryland, USA; NeoImmuneTech, Inc., Rockville, Maryland, USA; Fred Hutchinson Cancer Center, Seattle, Washington, USA; Sidney Kimmel Comprehensive Cancer Center at Johns Hopkins, Baltimore, Maryland, US

**Keywords:** high grade glioma, treatment related lymphopenia, NT-I7

## Abstract

**Background:**

High-grade gliomas (HGG) have a poor prognosis despite aggressive treatment. Severe, persistent lymphopenia occurring in HGG patients after concurrent chemoradiation is associated with worse survival. NT-I7, a long-acting interleukin-7 analog, has been shown to increase CD4 and CD8 counts in healthy, septic, and HIV-positive adults. This multi-institutional, NCI-funded dose-escalation trial is the first to evaluate NT-I7 safety and activity in HGG patients with severe treatment-related lymphopenia (TRL) and the effect of co-administered glucocorticoids.

**Methods:**

Eligible HGG patients had CD4 counts <300 cells/mm^3^ after 5 weeks of standard chemoradiation and were receiving either ≤0.75 or ≥4 mg/day of dexamethasone. Patients received a single intramuscular dose of NT-I7 (60 or 360 µg/kg) post-chemoradiation, followed by safety evaluation and multi-parameter, longitudinal monitoring of lymphocyte populations and immunologic function.

**Results:**

NT-I7 was well tolerated in all 12 patients (median age 64; median CD4 count 161 cells/mm³) before the study closed prematurely. Absolute lymphocyte counts doubled in 83% (10/12; 95% CI: 51.6%-97.9%) of patients, and CD4 counts doubled in 42% (5/12; 95% CI: 15.2%-72.3%) of patients. Glucocorticoid use did not significantly affect CD4 or lymphocyte increases. Correlative immune profiling revealed increased Ki67 expression in CD4 (*P* < .005) and CD8 (*P* < .05) after one week, along with the expansion of CD4 and CD8 T-cell subsets and CD56 + natural killer cells.

**Conclusions:**

NT-I7 is well tolerated and effectively increases lymphocyte and CD4 counts in severe TRL patients, regardless of glucocorticoid use, suggesting its potential to mitigate TRL and improve outcomes in HGG.

**Key Points**

- NT-I7 was safe, well tolerated in high grade glioma patients with treatment-related lymphopenia.
- NT-I7 increased absolute lymphocyte count and CD4 counts.
- Glucocorticoid use did not measurably affect NT-I7 treatment-mediated CD4 or lymphocyte increases.

**Importance of the Study**

Severe treatment related lymphopenia (TRL) occurs in more than 40% of patients with high-grade gliomas (HGG) and is associated with reduced survival and poor immunotherapy response. IL-7 is a cytokine essential for T-cell survival and function. Low endogenous IL-7 levels can be found in HGG patients with TRL. This is the first study of NT-I7, a long-acting IL-7 analog, in patients with severe TRL. After one intramuscular injection of NT-I7, 83% of treated patients doubled their total lymphocyte counts, and 42% doubled CD4 counts. Correlative immune profiling revealed increased Ki67 expression in CD4 and CD8 cells and expansion of CD4 and CD8 T-cell subsets and CD56 + NK cells. NT-I7 has the potential to mitigate or reverse severe TRL in patients with HGG.

High-grade gliomas (HGG) are the most prevalent primary malignant brain tumors in adults. After first recurrence, the median overall survival for patients with HGG ranges 5.5-12.6 months, highlighting the aggressive nature of these tumors.^1^ HGGs have been particularly resistant to standard chemotherapeutic agents, targeted agents, tumor treating fields, and immunotherapies. Furthermore, treatment efforts are hindered by challenges such as poor drug delivery across the blood-brain barrier,^2^ an immunosuppressive tumor microenvironment,^3^ and profound and sustained treatment-related lymphopenia (TRL), which impairs optimal anti-tumor responses.^4^

Severe TRL occurs in about 40% of patients with newly diagnosed HGG after standard radiation and chemotherapy and can persist for over a year.^5^ Although these patients routinely receive 3 lymphotoxic therapies—corticosteroids, temozolomide, and focal radiation—TRL appears to be primarily due to inadvertent, deleterious effects of radiation on circulating lymphocytes. In mouse models, focal radiation to the brain results in a rapid decrease in total lymphocyte counts regardless of radiation schedule or mouse strain.^6^ Lymphocytes are highly radiosensitive, and simulations suggest that each radiation fraction kills about 6% of circulating lymphocytes.^7^ A similar impact of radiation on circulating lymphocytes has also been observed in patients with multiple other malignancies who did not receive either temozolomide or glucocorticoids.^8^ In patients with HGG and other solid tumors, such as non-small cell lung cancer,^9^ pancreatic cancer,^10^ head and neck cancer,^11^ esophageal cancer,^12^ TRL is independently associated with poorer survival due to disease progression.^8,13-15^ Correction of TRL has been shown to improve survival in glioma animal models.^16^ In addition, lymphopenia caused by radiation is linked to a poor response to immunotherapy.^17^

Patients with severe TRL have inappropriately low levels of interleukin-7 (IL-7), a non-redundant homeostatic cytokine critical for T-cell survival and function.^18,19^ IL-7 is essential for the survival, proliferation, and homeostasis of T cells, including CD4 and CD8 memory T cells,^20,21^ and has been clinically shown to increase CD4 T-cell counts in patients with HIV,^22^ and sepsis.^23^ NT-I7 (efineptakin alfa, rhIL-7-hyFc) is a long-acting IL-7 analogue that consists of recombinant human IL-7 and a hybrid Fc (hyFc) region, which extends its half-life.^24^ NT-I7 has also been shown to induce an inflamed tumor microenvironment, an appealing attribute given the immunosuppressive nature of gliomas.^20,21,25,26^ Notably, the biological activity of NT-I7 on lymphoid cells is systemic and does not require penetration of the blood-brain barrier to impact lymphocyte counts.^27^

Animal models and human trials in healthy individuals suggest that NT-I7 safely increases T-cell counts. In glioma-bearing mice, NT-I7 administered after radiation and temozolomide resulted in increased CD4 and CD8 T-cell counts and improved survival.^16^ In healthy adults, NT-I7 was well tolerated^28^ and markedly increased CD4 and CD8 T-cell counts while broadening T-cell receptor diversity.^29^ More recently, a compassionate-use case series with NT-I7 demonstrated increased total lymphocyte counts in 16 of 18 glioblastoma patients undergoing chemotherapy.^30^

Given the low IL-7 levels found in patients with severe TRL following standard therapy for HGG and its association with survival, a phase I study with NT-I7 was conducted in adults with newly diagnosed HGG who developed severe TRL after concurrent radiation and temozolomide. Despite the recognized association between TRL and poor outcomes, there are currently no effective strategies to mitigate TRL in patients with HGG. Therefore, investigating NT-I7 as a means to restore lymphocyte counts addresses a critical unmet need in the management of these patients. The primary objectives were to determine the maximum tolerated dose (MTD) of NT-I7, to evaluate the CD4 and lymphocyte levels after NT-I7 treatment, and to evaluate whether the concurrent use of dexamethasone would interfere with the NT-I7-driven CD4 and lymphocyte expansion.

## Methods

### Study Information

This was a joint study of the National Cancer Institute (NCI)-funded Adult Brain Tumor Consortium (ABTC) and the Cancer Immunotherapy Trials Network (CITN) and included multiple centers across the United States. The institutional review board of each ABTC participating institution approved this study. This trial was registered with ClinicalTrials.gov (NCT02659800).

### Primary Objectives

The primary objective of this study was to determine the MTD or an optimal biological dose (OBD) of NT-I7 in HGG patients with severe lymphopenia.

### Secondary Objectives

Several secondary objectives were to evaluate the effects of NT-I7 in this patient population. Specifically, to: i) evaluate the OBD of NT-I7; ii) evaluate the effect of concurrent dexamethasone; iii) evaluate the duration of the NT-I7 effect on CD4 T-cell counts over time; iv) evaluate the absolute lymphocyte count (ALC) over time and serial T-cell subtypes; v) evaluate serial cytokine levels; vi) evaluate the impact of adjuvant temozolomide on NT-I7 effects on CD4 T-cell counts; vii) evaluate anti-drug antibodies (ADA); viii) evaluate the pharmacokinetic profile of NT-I7; and ix) evaluate the safety and toxicity of NT-I7 in patients with HGG.

### Eligibility Criteria

Patients eligible for this study were required to be ≥18 years of age with histologically confirmed HGG by pathology (WHO grade 3/4) who had undergone surgical resection with post-operative treatment that included at least 80% of standard radiation and concomitant temozolomide. In addition, their CD4 T-cell counts were required to be ≤ 300 cells/mm^3^ in the last 7 days of standard therapy (severe TRL), and a Karnofsky Performance Status (KPS) ≥ 60% was required. Severe TRL was prospectively defined according to CTCAE v5.0. Grade 3 lymphopenia corresponds to an ALC < 500 cells/mm³ (or CD4 < 200 cells/mm³) and Grade 4 to an ALC < 200 cells/mm³ (or CD4 < 50 cells/mm³). To facilitate accrual, an IRB-approved protocol amendment allowed enrolment of patients whose CD4 count was  < 300 cells/mm³, provided they still met the CTCAE grade 3/4 ALC criterion. Two separate cohorts were structured to determine if glucocorticoids affected the ability of NT-I7 to improve CD4 T-cell counts. The first cohort received physiologic doses (0.75 mg) of dexamethasone/day, while the second cohort received therapeutic doses defined as ≥ 4 mg of dexamethasone/day. Patients receiving between 0.75 and 4 mg of dexamethasone per day were not eligible for this study. Exclusion criteria also included prior chemotherapy, immunotherapy, or biologic agents; use of other investigational agents; uncontrolled intercurrent illness; known or screening-period-determined QTc interval > 450 msec; and autoimmune diseases. All patients provided written consent.

### Study Design and Treatment Plan

**Figure 1.**
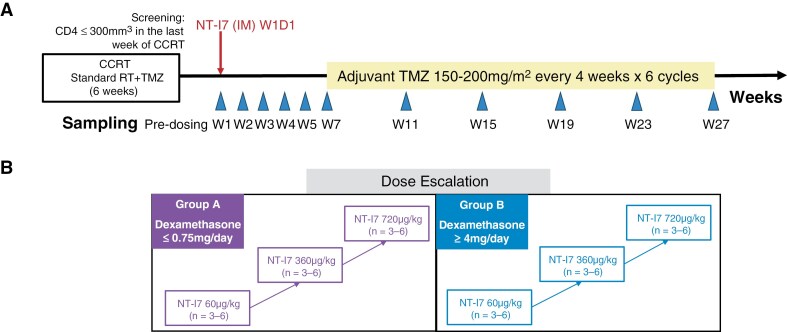
Treatment schema (panel A) and dose-escalation plan (panel B). Abbreviations: CCRT = chemoradiotherapy; RT = radiotherapy; TMZ = temozolomide; IM = intramuscular; W = week; D = day.

This phase I trial used a modified 3 + 3 design with 3-6 patients per dose cohort. Dose escalation and safety were evaluated independently in patients who were taking physiologic doses of dexamethasone (Group A, ≤0.75 mg/day) and in patients taking relatively high doses of dexamethasone (Group B, ≥4 mg/day) (Figure 1). A single dose of NT-I7 (efineptakin alfa, rhIL-7-hyFc) was administered one week after completion of concurrent RT + TMZ and a pre-planned 6-week interval was observed before the start of adjuvant TMZ. This interval (2 weeks longer than the 4-week STUPP standard) was incorporated to allow the full pharmacodynamic effect of a single NT-I7 dose—which peaks at ~6 weeks—to be evaluated without the confounding lymphotoxicity of TMZ. All patients received NT-I7 by intramuscular (i.m.) injection. The starting dose was 60 µg/kg, which had demonstrated efficacy in increasing lymphocyte counts in a phase I clinical study in normal healthy volunteers.^28^ Dose escalations were planned in a stepwise fashion using 3 pre-specified dose levels of NT-I7 (60 µg/kg, 360 µg/kg, 720 µg/kg). NT-I7 was administered at 2 dose levels: 60 µg/kg and 360 µg/kg. The highest planned dose level of 720 µg/kg was not tested due to early study closure.

### PBMC Isolation

Whole blood was collected into acid citrate dextrose (ACD) vacutainers at specified time points for the collection of peripheral blood mononuclear cells (PBMC). Samples were collected and shipped ambiently to the Cancer Immunotherapy Trials Network’s (CITN) Central Immune Monitoring Laboratory (CIML) so that the sample was received and processed within 24 hours of blood draw. PBMCs were isolated using Ficoll-Hypaque and cryopreserved as previously described.^31^

### Flow Cytometry

#### Whole blood immunophenotyping.

The change in absolute numbers, frequencies, and phenotypes of T cells and other PBMC subsets was evaluated by flow cytometry using whole blood received within 24 h of blood draw at the CIML.

To assess the absolute numbers of the different lymphocyte populations in the periphery, whole blood collected in ACD vacutainers was stained (100 μL) in Trucount tubes (BD 340334) with a validated 21-color antibody panel staining mix containing 1 × Brilliant Stain Buffer (BD 563794), FACS Buffer (2% FBS in DPBS (Gibco 14190144)) and the following previously titrated antibodies: CD3 BUV805 (BD 612895), CD4 BUV661 (BD 612962), CD8 BUV496 (BD 564804), CD11c AF700 (BD 561352), CD14 BV510 (BioLegend 301831), CD16 BUV737 (BD 612786), CD19 BUV563 (BD 565697), CD25 BB515 (BD 565096), CD28 B700 (BD 745905), CD45 BUV395 (BD 563792), CD45RA VioGreen (Miltenyi Biotec 130-113-369), CD56 AF647 (BioLegend 362513), CD123 BV650 (BioLegend 306019), CD127 PE-Cy5 (BioLegend 351324), CD152 BV786 (BD 563931), CD197 APC/Fire 750 (BioLegend 353246), CD223 PE (BioLegend 369306), CD279 PE-Cy7 (BD 561272), CD366 BV421 (BioLegend 345007), HLA-DR BV605 (BioLegend 307640), TIGIT PE-D564 (BioLegend 372716). 900 μL of 1 × FACS Lysing Buffer (BD 349202) was added to stained samples for 15 min before freezing at -80 °C. Thawed samples were collected in batches on a BD Symphony A1 and analyzed using FlowJo v10.8 software (BD Life Sciences).

### Ki67 Staining

To assess the proliferation status of T cells and natural killer (NK) cells, Ki67 intracellular protein expression was measured using cryopreserved PBMCs. PBMCs were thawed in RPMI 1640 supplemented with 10% fetal bovine serum, 2 mM L-glutamine, 100 units/mL penicillin-streptomycin, and 50 U/mL benzonase. Cellular viability was evaluated using Fixable Viability Dye eFluor 780 (eBioscience, 65-0865-14) prior to permeabilization (FoxP3/Transcription Factor Staining Buffer Set, eBioscience, 00-5523-00) and application of fluorescently labeled antibodies CD8 PerCP-Cy5.5 (BD Biosciences, 341051), CD14 BV421 (BD Biosciences, 563743), CD56 BV650 (BD Biosciences, 564057), CD3 FITC (BioLegend, 344804), CD4 PE (BioLegend, 344606), and Ki67 (eBioscience, 17-5699-42). After fixation with 2% paraformaldehyde, samples were analyzed on a Becton Dickinson LSR Fortessa flow cytometer, and flow cytometry results were analyzed using FlowJo software v10.8 (BD Life Sciences).

### Cytokine Analysis

A panel of cytokines, chemokines, and growth factors was evaluated in serum collected at baseline and longitudinally after treatment. The concentrations of G-CSF, GM-CSF, IFNα2, IFNγ, IL-10, IL-12p70, IL-13, IL-15, IL-17A, IL-1β, IL-2, IL-3, IL-4, IL-5, IL-6, IL-7, IL-8, IP-10, MCP-1, TNF, CXCL9, and CXCL11 were quantified using Millipore’s Human Cytokine/Chemokine/Growth Factor Panel A (Millipore Sigma, Germany) according to the manufacturer’s instructions. Four analytes (GM-CSF, IFNα2, IL-17A, and IL-3) were consistently under the lower limit of detection (LLOQ) and were excluded from the analysis. The dataset was scaled and centered for each marker. The scaled dataset was then hierarchically clustered and plotted as a heatmap in R.

### Clinical Laboratory Tests

Clinical laboratory tests consisted of a complete blood count (CBC) with differential, including an ALC, white blood cell count (WBC), absolute neutrophil count (ANC), hemoglobin, hematocrit, and platelet count assessed weekly. CD4 T-cell count was measured before starting NT-I7, and then at week 1, week 3, week 7, and monthly thereafter.

### Immunogenicity Analysis

Immunogenicity was measured using a risk-based, tiered bridging electrochemiluminescence enzyme-linked immunosorbent assay (ELISA)-based testing approach. The assay included a core assay comprising screening, confirmatory, and titer testing for detecting anti-drug antibodies (ADA) to NT-I7 and an IL-7 epitope-specific assay. ADA-positive patient serum samples were further tested for the presence or absence of neutralizing ADA (NADA) using a cell-based assay.

### Statistical Considerations

This was a multicenter, non-randomized, open-label Phase I trial, followed by a randomized pilot study to evaluate the effect of NT-I7 (efineptakin alfa, rhIL-7-hyFc) on CD4 T-cell counts. In Phase I, 3 pre-specified doses (60, 360, and 720 µg/kg) were planned to be tested in Groups A (dexamethasone ≤.75 mg/day) and B (dexamethasone ≥4 mg/day) independently. A modified 3 + 3 design with 3-6 patients per dose cohort was used for the safety evaluation. Dose escalation took place in a stepwise fashion with a targeted DLT level of less than 33%. The safety evaluation period was 4 weeks after NT-I7 injection. If an MTD was not reached in the 3 prespecified doses, the dose chosen for the pilot study was to be determined based on its optimal biological activities. A minimum of a 2-fold increase of CD4 counts was required to declare an optimal biologic dose. The 2-fold increase was expected within 6 weeks after the NT-I7 injection without any concomitant chemotherapy. Such an increase required to be sustained to Week 7.

The trial was open to enrollment in December 2018 and terminated early for administrative reasons in October 2023. A total of 15 patients were enrolled in the Phase I portion of the trial. Twelve of the patients were treated with 2 pre-specified doses of NT-I7 (60 and 360 μg/kg). The 720 μg/kg dose level was not tested. The baseline patient and disease characteristics were summarized using descriptive statistics. The safety data were collected during patients’ clinical visits or through telemedicine during the COVID period. The safety information among the 12 treated patients was evaluated based on NCI Common Terminology Criteria for Adverse Events (CTCAE v.5), and the incidence rate of adverse events was presented using a proportion. Some protocol-specified laboratory measurements were missed due to COVID restrictions, especially on CD4 counts. The 2-fold increase in CD4 counts during the first 6 weeks of the trial was unevaluable due to the large percentage of missing data. A 2-fold increase in CD4 counts at any time point during the trial was calculated instead. No imputation was made to the missing data. However, the patients’ standard complete blood counts with differentials were collected while they were receiving chemotherapy. The proportion of patients who reached a 2-fold increase in CD4 counts or in ALC at any time point during the trial was estimated using the binomial distribution along with a 95% confidence interval. The absolute laboratory value changes from the baseline were presented using descriptive statistics. The correlative studies were exploratory and mechanistic in nature, and the data were presented descriptively. The analysis of clinical data was performed using SAS, version 9.4 (SAS Institute, Cary, NC).

## Results

### Patient Characteristics and Study Flow

A total of 15 patients with HGG were enrolled in the study between December 2018 and November 2021 (**Supplementary Figure S1**). The trial was terminated early for administrative reasons in October 2023. Among the 15 enrolled patients, 12 patients received NT-I7, and their data are presented below (**Table 1**). Three patients who signed consent forms never received NT-I7 due to withdrawal of consent prior to treatment initiation (*n* = 1), rapid disease progression requiring alternative interventions (*n* = 1), and discovery of ineligibility after consent (*n* = 1). One of these patients did not have baseline information collected and thus their information is not included in Table 1. Among the 12 NT-I7 treated patients, the median age was 62.4 years (range: 38.8-71.3). Eleven of the 12 (92%) patients were IDH wild-type, and 9 (75%) had glioblastoma. Only 25% of the patients were MGMT methylated. Given the small cohort size (*n* = 12) and the low frequency of MGMT-methylated tumors, no meaningful trend between MGMT status and NT-I7-induced lymphocyte recovery could be discerned. Seventy-five percent underwent gross total or partial resection, with the remaining 25% (3 patients) receiving biopsy only. These patients were 75% white, 67% male, and 58% had a Karnofsky performance status between 90 and 100. As per the eligibility criteria, all patients were severely lymphopenic at study entry, with median absolute lymphocyte counts of 350 cells/mm^3^ (range 200-600) and a median CD4 count of 161 cells/mm^3^ (range 106-260).

**Table 1.** Patient and disease baseline characteristics

Patient Baseline Characteristics	Subgroup Treated Patients (*n* = 12)	All Patients (*N* = 14)
Age: bedian year (range)	62.4 (38.8-71.3)	62.4 (38.8-71.3)
Race:		
White: No. (%)	9 (75.0)	11 (78.6)
Gender: male no. (%)	8 (66.7)	10 (71.4)
KPS:		
90-100: No. (%)	7 (58.3)	9 (64.3)
70-80: No. (%)	5 (41.7)	5 (35.7)
MGMT: No. (%)		
Methylated:	3 (25.0)	3 (21.4)
Unmethylated:	8 (66.7)	8 (57.1)
Unknown:	1 (8.3)	3 (21.4)
IDH- wildtype: no. (%)	11 (91.7)	13 (92.9)
Radiation therapy: no. (%)	12 (100)	14 (100)
Steroids dose:		
Group A (*n*=7)	.5 QD (*n*=1)	.5 QD (*n*=1)
Group B (*n*=5)	4 BID QD (*n*=4)	4 BID QD (*n*=4)
	2 BID (*n*=1)	2 BID (*n*=3)
CD4 counts: median (range)		
Group A (*n*=7)	210 Cells/µL (110-260)	
Group B (*n*=5)	155 Cells/µL (106-196)	
Overall (*n*=12)	161 Cells/µL (106-260)	
Absolute lymphocyte count:		
Median (range)	350 Cells/µL (200-600)	
Anticonvulsant		
Yes: No. (%)	8 (66.7)	10 (71.4)
Histology prior to stud		
Glioblastoma multiforme: no. (%)	9 (75.0)	11 (76.6)
A naplastic astrocytoma: no. (%)	3 (25.0)	3 (21.4)
Surgery		
Biopsy: no. (%)	3 (25.0)	4 (28.6)
Subtotal resection: no. (%)	6 (50.0)	6 (42.8)
Gross total resection: no. (%)	3 (25.0)	4 (28.6)
Prior surgery: median (range)	1 (1-2)	1 (1-2)

Patients were assigned to Group A if they were receiving physiologic doses of dexamethasone (≤0.75 mg/day) or Group B if they were receiving therapeutic doses (≥ 4mg) of dexamethasone per day. A total of 7 patients were enrolled in Group A. Six of these patients were not receiving any supplemental steroids, and one was receiving dexamethasone at .5 mg/day. A total of 5 patients were assigned to Group B. Three of these patients were receiving dexamethasone at 4 mg/day, two 8 mg/day, and one 12 mg/day (**Table 1**).

NT-I7 was administered at 60 µg/kg and 360 µg/kg. The highest dose level of 720 μg/kg was not tested due to pre-mature study closure. In group A, 4 patients received 60 µg/kg and 3 patients received 360 µg/kg. In Group B, 3 patients received 60 µg/kg and 2 patients received 360 µg/kg (**Supplementary Figure S1**).

### NT-I7 Shows Minimal Toxicity

There was no dose-limiting toxicity observed at the 2 dose levels in either Group A or Group B. Treatment-related adverse events are presented in **Table 2**. Only one patient in Group A (60 µg/kg) experienced a Grade 3 maculopapular rash, possibly related to NT-I7. The rash resolved spontaneously within 7 days without specific intervention.

**Table 2.** Treatment-related adverse events

Group	Dose Level	Adverse Event	Grade	*N* (%)
A	60	Fatigue	2	1 (8.3%)
A	60	Rash maculo-papular	3	1 (8.3%)
A	60	Rash maculo-papular	1	1 (8.3%)
A	360	White blood cell decreased	1	1 (8.3%)
A	360	Fever	1	1 (8.3%)
A	360	Pain	1	1 (8.3%)
B	360	Alanine aminotransferase increased	1	1 (8.3%)

### Primary Objective

The MTD could not be determined as per protocol, as the highest pre-specified dose level (720 µg/kg) was not administered due to the premature closing of this study within the ABTC.

### Absolute Lymphocyte Counts (ALC) After NT-I7 Treatment

**Figure 2.**
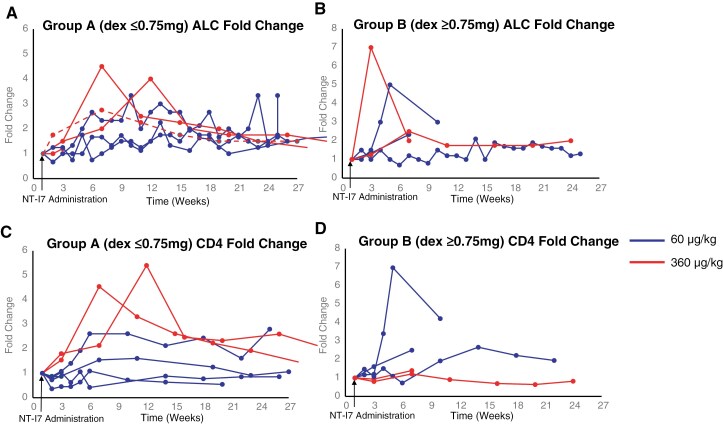
(A-B) Fold-change of absolute lymphocyte count (ALC) over time; (C-D) fold-change of CD4⁺ T-cell count over time. Solid line = patients receiving 60 µg/kg; dashed line = 360 µg/kg. NT-I7 was administered at Week 1.

ALC counts rose in all 12 patients after NT-I7 administration (12/12, 95% CI: 73.5-100%) during the trial period (**Figure 2A, B**; **Supplementary Figure S2A**, **B**). In addition, ALC counts more than doubled in 83% (10/12, 95% CI: 51.6-97.9%) of patients during the trial period. In Group A (dexamethasone ≤ .75 mg/day), the ALC median fold change was 1.13 (range 0.8-1.5, *P* = .24) at 3 weeks and 2.33 (range .75-2.67, *P* = .06) at weeks 6 to 7. In Group B (dexamethasone ≥ 4 mg/day), the ALC median fold change was 1.33 (range 1.0-7.0, *P* = .17) at week 3 and 2.17 (0.7-2.33, *P* = .38) at week 6 to 7. While this study was not powered or designed for a direct comparison between Groups A and B, the similarity in lymphocyte rises in Groups A and B at 3 and 6 weeks suggests that dexamethasone did not impact the effect of NT-I7 on increasing ALC in these patients. In addition, with the small sample sizes in this study, we did not observe any major differences in ALC counts between the 60 µg/kg and 360 µg/kg dose levels. A higher dose (720 µg/kg) was not tested due to the early closure of this trial.

### CD4 T-Cell Counts After NT-I7 Treatment

CD4 counts rose in 91% of patients (11/12, 95%CI: 73.5%-100%) (**Figure 2C, D**), and the CD4 counts more than doubled in 42% (5/12, 95% CI: 15.2%-72.3%) of patients during the trial period. Although NT-I7 increased CD4 T cells, the median increase was not statistically significant compared to baseline for either group (**Figure 2C, D**; **Supplementary Figure S2C**, **D**). In Group A (dexamethasone ≤ 0.75 mg/day), CD4 T-cell counts increased a median of 1.06-fold (range .45-1.54, *P* = .47) at 3 weeks and a median of 1.83 (range: 0.42-2.61, *P* = .15) at 6–7 weeks (Figure 2C). In Group B (dexamethasone ≥ 4 mg/day), CD4 T-cell counts increased a median of 1.07-fold (range 0.80-1.62, *P* = .47) at 3 weeks and a median of 1.31-fold (range 0.73-2.5, *P* = .54) at 6–7 weeks (**Figure 2D**). Similarly to ALC results, we did not observe any obvious difference in CD4 T-cell counts between Group A and Group B, suggesting the effects of NT-I7 at this quite low dose do not appear greatly affected by steroid use. These analyses are limited by the small sample sizes in this phase I study and the limited number of CD4 counts due to restrictions on research laboratory testing during the COVID-19 pandemic.

### T cell and NK Proliferation in Response to NT-I7 Treatment

**Figure 3.**
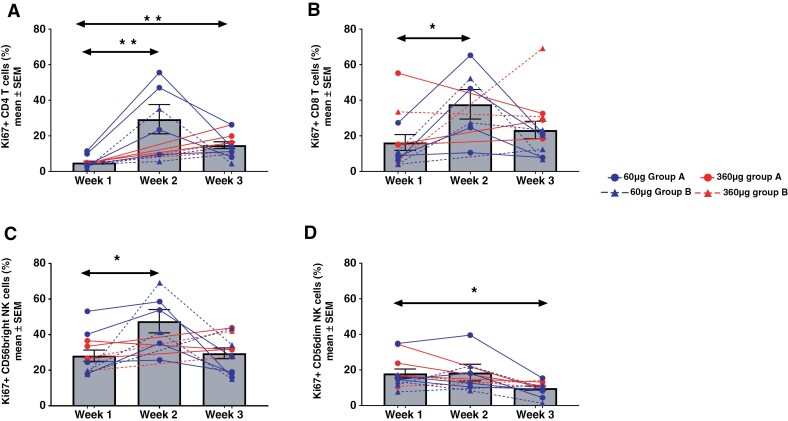
Proliferation of peripheral-blood lymphocytes after a single dose of NT-I7. Proliferation was assessed in cryopreserved PBMC. CD4⁺, CD8⁺, and CD56^bright NK cells showed a statistically significant increase in Ki-67 expression after NT-I7 (panels A, B, & C, respectively); CD56^dim NK cells (panel D) did not. Type 2, 2-tailed tests were used (* *P* < .05; ** *P* < .005). Each patient is represented by a unique colour. Solid lines = 60 µg/kg; dashed lines = 360 µg/kg.

**Figure 4.**
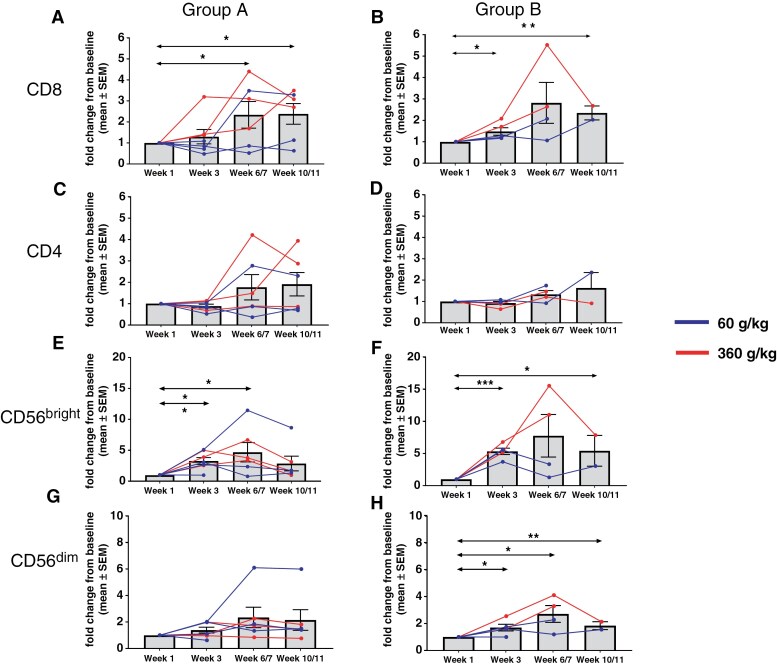
Single-dose NT-I7 expands peripheral CD4⁺ and CD8⁺ T cells and NK-cell subsets. Patients were stratified by dexamethasone (Dex) use: ≤ .75 mg/day (Group A: panels A & C, E & G) vs ≥ 4 mg/day (Group B: panels B & D, F & H). Shown are peripheral-blood CD4⁺ T-cell counts (panels A, B), CD8⁺ T-cell counts (panels C, D), CD56^bright NK cells (panels E, F), and CD56^dim NK cells (panels G, H). Each patient is designated by a unique colour. Solid lines = 60 µg/kg; dashed lines = 360 µg/kg. Type 2, 2-tailed tests were performed (* *P* < .05; ** *P* < .005; *** *P* < .00005).

Exploratory analyses were aimed at further understanding the impact of NT-I7 administration on the systemic changes of the different immune subsets. Ki67 expression was analyzed on PBMCs as a marker of proliferation. Due to sample availability, only 6 subjects (6/12, 50%) were included in the week 2 analysis. Combination of samples from Group A and Group B show transitory but significant upregulation of Ki67 expression as a direct result of NT-I7 administration in both CD4 and CD8 T cells at week 2, with expression levels almost returning to baseline by week 3 (**Figure 3A, B**). This result appears independent of the concomitant use of steroids, though the analysis was limited by the sample size. CD56^bright^, but not CD56^dim^, NK cells also significantly increased Ki67 expression (**Figure 3** C, D). Consistent with the Ki67 observations, the numbers of peripheral CD8 T cells significantly increased, with peak expansion measured around week 6, regardless of the use of concomitant steroids (**Figure 4** A, B). Peripheral CD4 T cells also reached peak expansion by week 6, but the increase was not statistically significant, potentially due to the low sample size (**Figure 4** C, D). This suggests that NT-I7 may have a stronger effect on CD8 T cells in these types of patients. Similarly, in alignment with the Ki67 expression data, peripheral CD56^bright^ NK cells significantly expanded (**Figure 4** E, F). A small increase in CD56^dim^ NK cells was also observed in subjects with therapeutic dexamethasone treatment (**Figure 4** G, H) despite this subset not showing increased Ki67 expression. Interestingly, an increase in B cells in the group with low dexamethasone administration was observed (**Supplementary Figure S3**). Since peripheral B cells do not express the IL-7 receptor, this increase may likely be due to indirect mechanisms.

T-cell subset analysis showed that absolute numbers of CD8 T-cell subsets (central memory, effector memory, and terminal effectors) were increased with treatment, while their relative proportions remained unchanged (**Supplementary Figure S4**). Similar trends were observed for the CD4 T-cell subsets, although changes did not reach statistical significance (**Supplementary Figure S5**). All changes followed similar patterns in subjects with low or high dexamethasone treatment (data not shown).

### Inflammatory Cytokine Profiles

Detailed analysis of peripheral inflammatory cytokines and growth factors at baseline (week 1) and post-treatment time points did not show different signature profiles among groups (**Supplementary Figure S6**). Subjects treated with a low or high dose of dexamethasone had similar signature profiles at baseline and responded similarly to the treatment.

### NT-I7 Immunogenicity

Out of 61 samples tested from 12 subjects, treatment-emergent ADA was detected in 11 samples from 4 subjects. Samples from one subject in Group A failed QC, and the data were not reportable. Among the 11 ADA positive samples, only 1 showed neutralizing activity (W07 for a subject enrolled in the 60 µg/kg NT-I7 dose, high dexamethasone group). For the 4 (36.4%) subjects with emergent ADA, the first occurrence was between weeks 7 and 11. Two of the 4 subjects with detectable ADA had long-term follow-up, and, in both cases, the ADA antibodies were no longer detectable at week 23 (1 subject) or at week 27 (1 subject), suggesting that the ADA generated in these subjects is transient. Endogenous IL-7 levels post-NT-I7 treatment were not affected regardless of the presence of ADA (data not shown).

The limited number of patients included in this study led to challenges in assessing any potential relationship between ADA and pharmacodynamic responses. However, in this analysis, no clear relationship was observed.

## Discussion

This phase I trial is the first to assess the use of NT-I7 (efineptakin alfa, rhIL-7-hyFc), a long-acting IL-7, in HGG patients with severe TRL. A phase 1 study was essential as a safe dose in this population had not yet been established at the time of protocol initiation. These patients were severely lymphopenic following treatment with standard radiation and temozolomide for their newly diagnosed high grade gliomas. Severe TRL is common in patients with HGG after standard concurrent chemoradiotherapy and is associated with shorter survival due to tumor progression.^8^ In a prospective study of 96 patients with HGG, absolute lymphocyte and CD4 counts were relatively normal before therapy, and after 6 weeks of radiation and concurrent temozolomide, 73% of patients had CD4 counts that fell below 300 cells/mm^3^, and 40% had CD4 counts less than 200 cells/mm^3^.^13^ Such profound immunosuppression is consistent with the clinical recommendation for pneumocystis jirovecii pneumonia prophylaxis in this patient population.

Our results from the Phase I trial suggest that NT-I7 leads to increases in ALC and improves CD4 and CD8 T-cell proliferation in HGG patients with severe TRL. Encouragingly, CD8 T-cell counts were increased regardless of the steroid dose patients were receiving for their brain edema. Importantly, the efficacy of NT-I7 in increasing lymphocyte counts appeared to be unaffected by concurrent glucocorticoid use, although our sample size limited rigorous comparison. This is particularly relevant for HGG patients who often require steroids to manage cerebral edema, indicating that NT-I7 can be effectively administered without compromising its therapeutic benefits due to steroid interference. Randomized studies are needed to confirm the effect of NT-I7.

We also found that NT-I7 was correlated with peripheral CD56^bright^ NK-cell expansion. Since conventional NK cells do not express high levels of the IL-7 receptor (CD127), it is unclear whether this effect is directly or indirectly attributable to NT-I7 dosing. Our results are consistent with prior work that has suggested that IL-7 may enhance the survival of NK CD56^bright^ cells through increased expression of BCL-2.^32^ In vitro, NK cells lysed glioblastoma cells in a dose-dependent function. Moreover, inhibition of NK activity in xenograft mouse models led to glioblastoma metastasis, suggesting that NK cells may have a role in tumor suppression.^33^ Additionally, NT-I7 has been shown to significantly increase survival and reduce tumor burden in a xenograft mouse model with multiple myeloma treated with invariant NK cell based allogenic CAR-T (CAR-iNKT) therapy.^34^ Pre-clinical studies have explored the use of NK cell-based immunotherapy, with further research ongoing in glioblastoma.^35,36^

Given the presence of immunosuppressive regulatory T cells and myeloid-derived suppressor cells within the glioma microenvironment, future studies should explore whether NT-I7 can modulate these immune-suppressive elements and enhance anti-tumor immunity within the central nervous system. Addressing this could increase the efficacy of immunotherapies in this setting.

In addition, NT-I7 could have an important role in enhancing immunotherapy. In a retrospective study involving head/neck cancer patients, treatment response to PD-1 inhibitors was associated with pre-treatment ALC; patients with ALC < 600 cells/µL had shorter progression free survival with PD-1 therapy compared to the cohort with ALC ≥ 600 cells/µL.^36^ Similarly, shorter time to progression was found in lymphopenic patients with solid tumors who were treated with nivolumab or pembrolizumab anti-PD-1 antibodies.^17^ The consideration that immunotherapy may be potentiated by enhancing lymphocyte counts warrants further study.

Overall, our results suggest that NT-I7 could be an important and safe option for patients with severe TRL. Further studies are required to determine if this will provide a measurable clinical benefit. It would be valuable to investigate whether administration of NT-I7 prior to radiation therapy could prevent severe lymphopenia from developing, rather than attempting to reverse it post-therapy. This approach may maintain lymphocyte counts during the most critical period of the anti-tumor response. In addition, studies are needed to determine if the use of NT-I7 could be useful in reducing the risk of developing severe TRL in high-risk patients with brain and other cancers who will be treated with radiation therapy. Finally, it is appealing to consider NT-I7 as an adjuvant to cancer immunotherapy trials.

### Limitations

This study was severely impacted by the COVID-19 pandemic and by the premature closure of the ABTC. The small sample size and missing data due to COVID-19 restrictions limit the generalizability of our findings. Additionally, the lack of a control group prevents definitive conclusions about the efficacy of NT-I7 compared to the natural course of TRL recovery. However, the use of IL-7 has never before been explored in patients with severe TRL, and this trial accrued patients with a median CD4 count of 161 cells/mm^3^. As this was a phase I dose escalation study, there was no control arm documenting natural changes in ALC or CD4 counts over time without the administration of NT-I7. Further studies are needed to fully explore the potential of this novel therapeutic approach.

### Future Directions

Larger studies are needed to assess the use of NT-I7 to mitigate or prevent TRL in high-grade brain tumors and other systemic malignancies. NT-I7 has the potential to modulate immunotherapy. Future studies should also explore different dosing schedules and higher doses of NT-I7 than what were used in this study. Combination therapy with NT-I7 and immune checkpoint inhibitors may enhance T-cell activation and increase the efficacy of treatment. Combination treatment of pembrolizumab with NT-I7 for patients with recurrent GBM is currently under investigation (NCT05465954). Ongoing phase 2 trials are studying the combination of NT-I7 with checkpoint inhibitors for the treatment of other malignancies. In addition, the use of NT-I7 with chimeric antigen receptor T-cell (CAR-T) therapy has demonstrated promising results in mouse models.^24^ Combination therapy with immune modulating agents offers the potential to transform the treatment paradigm for patients with HGG.

## Conclusions

The long-acting interleukin-7 NT-I7 is safe and increases absolute lymphocyte counts and CD4 T-cell counts in patients with newly diagnosed high-grade gliomas with severe treatment-related CD4 lymphopenia. Its effect on lymphocyte counts does not seem to be affected by steroids.

## Supplementary Material

vdaf117_suppl_Supplementary_Figures_S1-S6

## Data Availability

De-identified data that support the findings of this study will be made available upon reasonable request and in accordance with our Data Management and Sharing Plan. Additional data or analyses not included in the published manuscript may be shared upon request, contingent on appropriate institutional and ethical approvals. Detailed protocols, metadata, and documentation (including case report forms, code used for analyses, and methodological workflows) will also be provided to ensure reproducibility, in line with NIH and journal data-sharing policies. Requests for access to original or third-party data analyzed in this article should be directed to the Principal Investigator, who will facilitate data sharing consistent with participant consents, privacy rights, and regulatory requirements.
