# Supplementary material for: Phase I study of NT-I7, a long-acting interleukin-7, in severe treatment-related lymphopenia following standard radiation and temozolomide for high-grade glioma
Source: Neurooncol Adv. 2025 Jun 7;7(1):vdaf117. doi: 10.1093/noajnl/vdaf117 (PMC12284640; doi:10.1093/noajnl/vdaf117)
Supplement: vdaf117_suppl_Supplementary_Figures_S1-S6 [file vdaf117_suppl_supplementary_figures_s1-s6.pptx]

## Slide 1
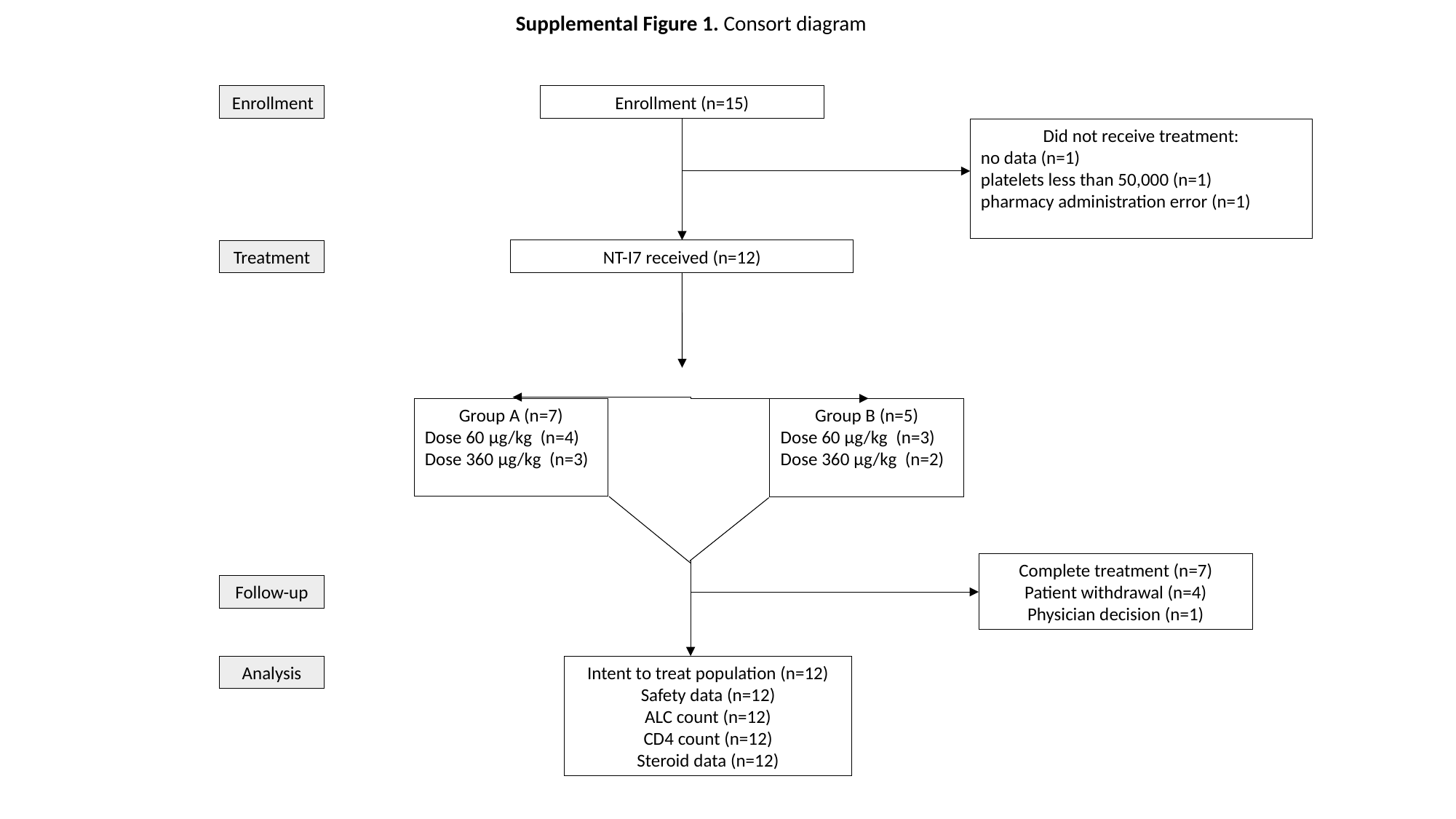

Supplemental Figure 1. Consort diagram
Enrollment
Enrollment (n=15)
Did not receive treatment:
no data (n=1)
platelets less than 50,000 (n=1)
pharmacy administration error (n=1)
NT-I7 received (n=12)
Treatment
Group A (n=7)
Dose 60 µg/kg (n=4)
Dose 360 µg/kg (n=3)
Group B (n=5)
Dose 60 µg/kg (n=3)
Dose 360 µg/kg (n=2)
Complete treatment (n=7)
Patient withdrawal (n=4)
Physician decision (n=1)
Follow-up
Analysis
Intent to treat population (n=12)
Safety data (n=12)
ALC count (n=12)
CD4 count (n=12)
Steroid data (n=12)

## Slide 2
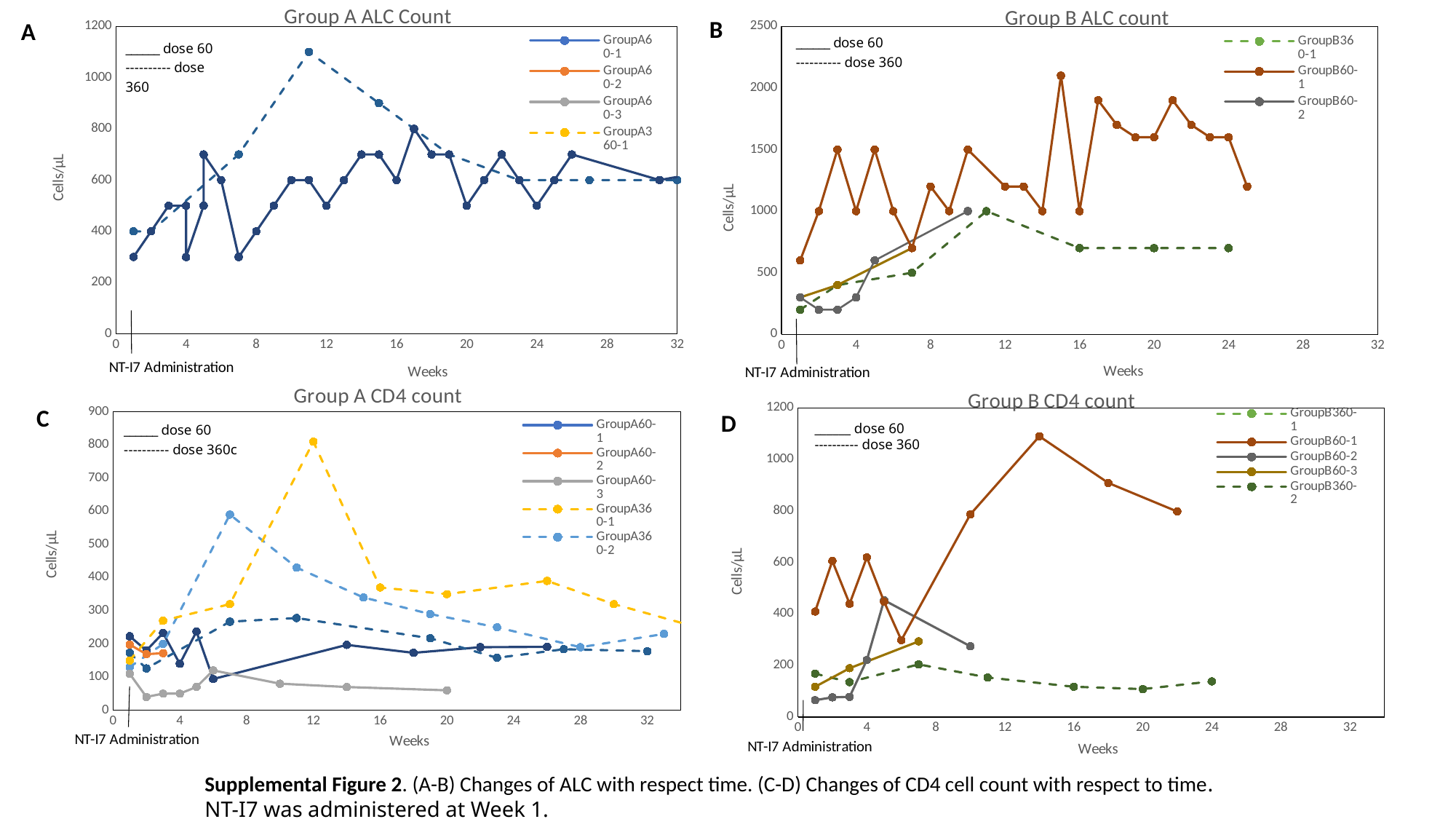

### Chart: Group B ALC count
| Category | GroupB360-1 | GroupB60-1 | GroupB60-2 | GroupB60-3 | GroupB360-2 |
|---|---|---|---|---|---|
### Chart: Group A ALC Count
| Category | GroupA60-1 | GroupA60-2 | GroupA60-3 | GroupA360-1 | GroupA360-2 | GroupA60-4 | GroupA360-3 |
|---|---|---|---|---|---|---|---|B
A
### Chart: Group B CD4 count
| Category | GroupB360-1 | GroupB60-1 | GroupB60-2 | GroupB60-3 | GroupB360-2 |
|---|---|---|---|---|---|______ dose 60
---------- dose 360
### Chart: Group A CD4 count
| Category | GroupA60-1 | GroupA60-2 | GroupA60-3 | GroupA360-1 | GroupA360-2 | GroupA360-3 | GroupA60-4 |
|---|---|---|---|---|---|---|---|C
D
Supplemental Figure 2. (A-B) Changes of ALC with respect time. (C-D) Changes of CD4 cell count with respect to time.
NT-I7 was administered at Week 1.

## Slide 3
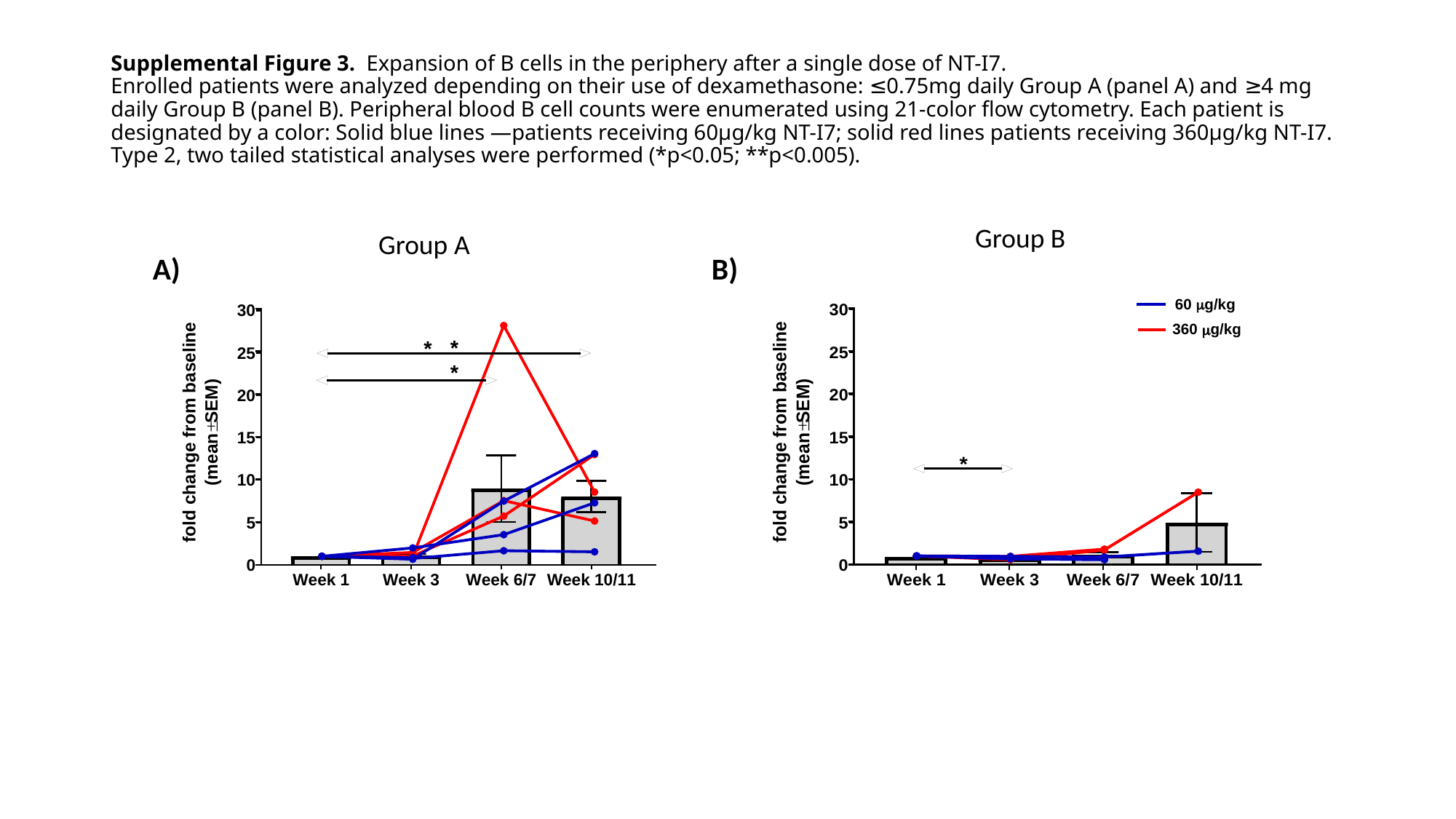

# Supplemental Figure 3. Expansion of B cells in the periphery after a single dose of NT-I7. Enrolled patients were analyzed depending on their use of dexamethasone: ≤0.75mg daily Group A (panel A) and ≥4 mg daily Group B (panel B). Peripheral blood B cell counts were enumerated using 21-color flow cytometry. Each patient is designated by a color: Solid blue lines —patients receiving 60µg/kg NT-I7; solid red lines patients receiving 360µg/kg NT-I7. Type 2, two tailed statistical analyses were performed (*p<0.05; **p<0.005).
Group B
Group A
B)
A)

## Slide 4
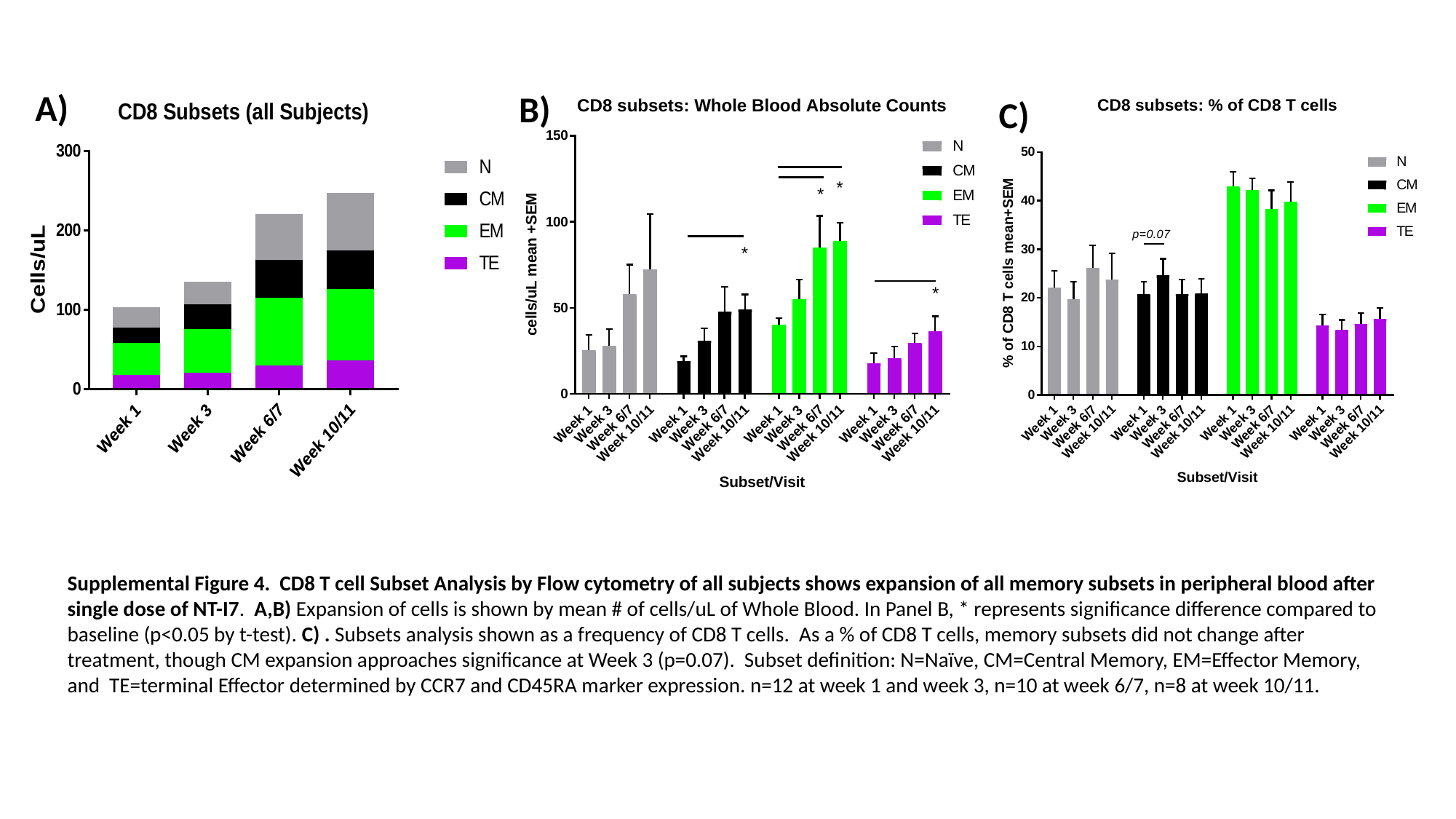

A)
B)
C)
Supplemental Figure 4. CD8 T cell Subset Analysis by Flow cytometry of all subjects shows expansion of all memory subsets in peripheral blood after single dose of NT-I7. A,B) Expansion of cells is shown by mean # of cells/uL of Whole Blood. In Panel B, * represents significance difference compared to baseline (p<0.05 by t-test). C) . Subsets analysis shown as a frequency of CD8 T cells. As a % of CD8 T cells, memory subsets did not change after treatment, though CM expansion approaches significance at Week 3 (p=0.07). Subset definition: N=Naïve, CM=Central Memory, EM=Effector Memory, and TE=terminal Effector determined by CCR7 and CD45RA marker expression. n=12 at week 1 and week 3, n=10 at week 6/7, n=8 at week 10/11.

## Slide 5
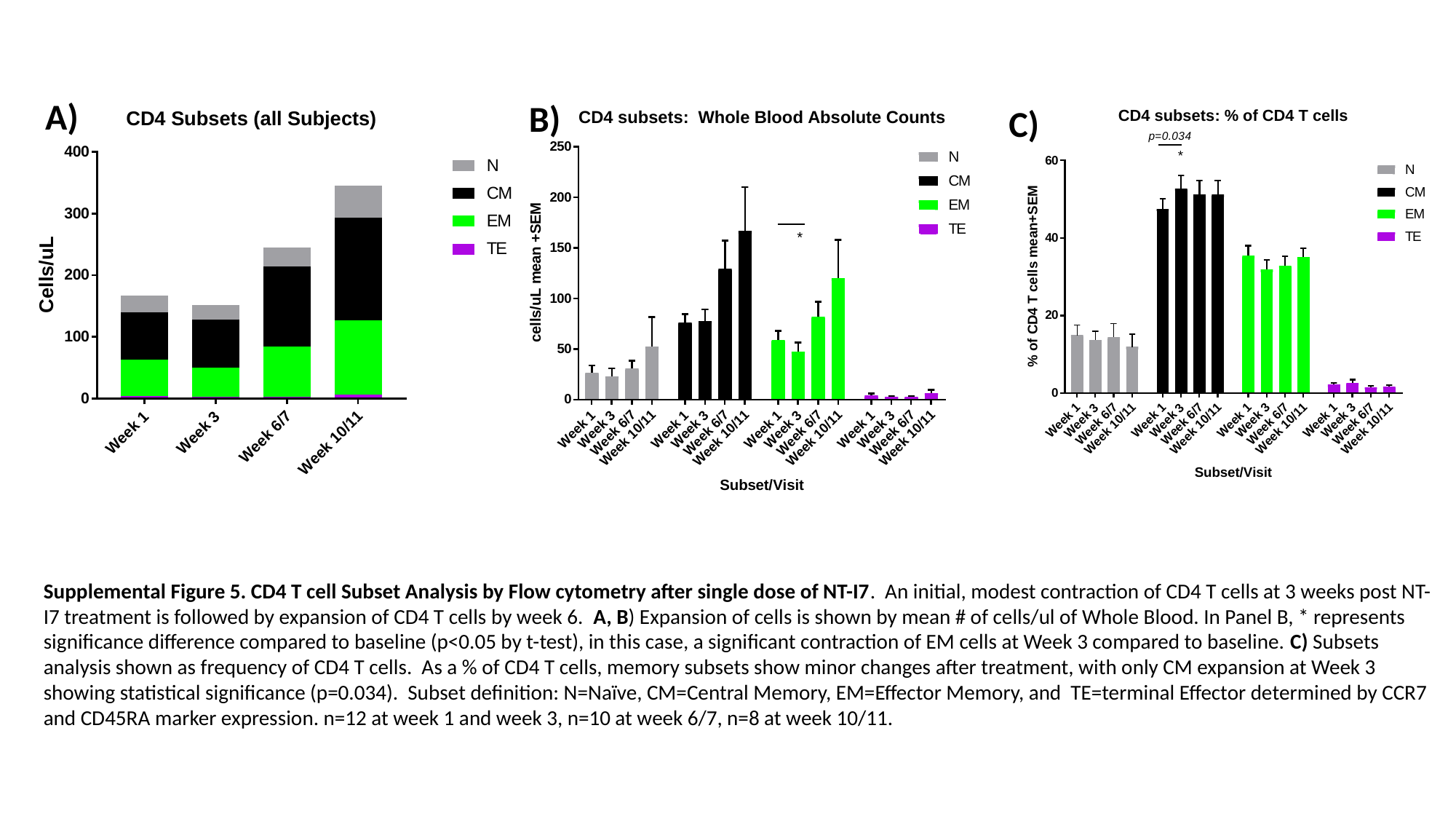

A)
B)
C)
Supplemental Figure 5. CD4 T cell Subset Analysis by Flow cytometry after single dose of NT-I7. An initial, modest contraction of CD4 T cells at 3 weeks post NT-I7 treatment is followed by expansion of CD4 T cells by week 6. A, B) Expansion of cells is shown by mean # of cells/ul of Whole Blood. In Panel B, * represents significance difference compared to baseline (p<0.05 by t-test), in this case, a significant contraction of EM cells at Week 3 compared to baseline. C) Subsets analysis shown as frequency of CD4 T cells. As a % of CD4 T cells, memory subsets show minor changes after treatment, with only CM expansion at Week 3 showing statistical significance (p=0.034). Subset definition: N=Naïve, CM=Central Memory, EM=Effector Memory, and TE=terminal Effector determined by CCR7 and CD45RA marker expression. n=12 at week 1 and week 3, n=10 at week 6/7, n=8 at week 10/11.

## Slide 6
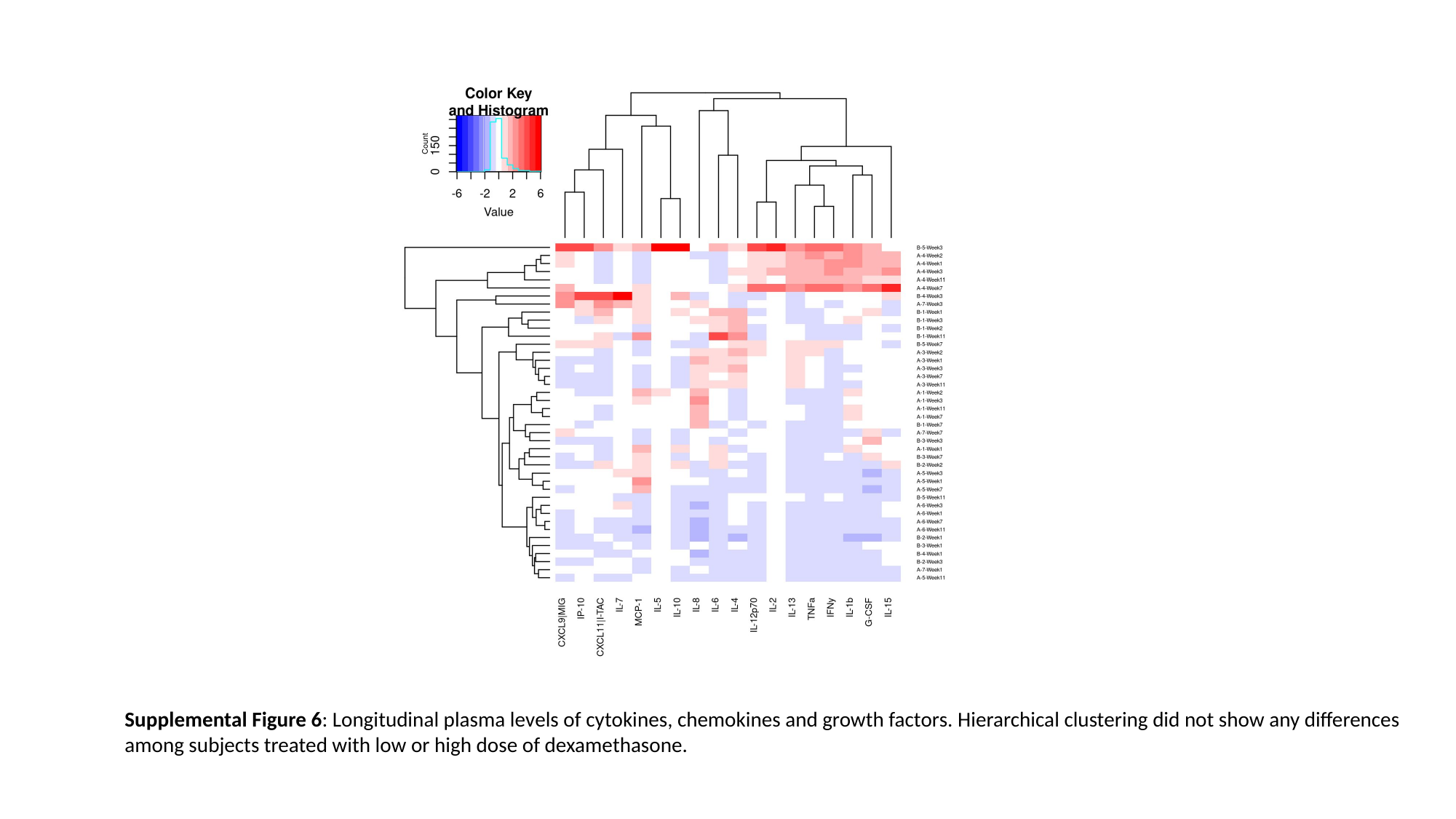

Supplemental Figure 6: Longitudinal plasma levels of cytokines, chemokines and growth factors. Hierarchical clustering did not show any differences among subjects treated with low or high dose of dexamethasone.
